# The acute response of tendon to loading: implications for rehabilitation

**DOI:** 10.1186/1757-1146-4-S1-I13

**Published:** 2011-05-20

**Authors:** Scott C Wearing, Nicole L Grigg, Sue L Hooper, Erin E Appleton, James E Smeathers

**Affiliations:** 1 Faculty of Health Sciences and Medicine, Bond University, Gold Coast, Qld, 4229, Australia; 2 Institute of Health and Biomedical Innovation, Queensland University of Technology, Brisbane, Qld, 4059, Australia; 3 Centre of Excellence for Applied Sports Science Research, Queensland Academy Sport, Nathan, Qld, 4111, Australia

## 

Achilles tendinopathy is a common disorder involving physically active and sedentary individuals alike. Although the processes underlying its development are poorly understood, tendinopathy is widely regarded as an ‘overuse’ injury in which the tendon fails to adapt to prevalent loading conditions. Paradoxically, there is emerging evidence that heavy eccentric loading of the Achilles tendon may be an effective conservative approach for treatment of tendinopathy, with success rates of 60–80% reported. Interestingly, loading exercises involving other forms of muscle action, such as concentric activation, have been shown to be less effective treatment options. However, little is known about the acute response of tendon to exercise at present, and there are few plausible explanatory mechanisms for the observed beneficial effects of eccentric exercise, as opposed to other forms of strain stimuli. This paper presents the findings from a series of experiments undertaken to evaluate the effect of various strain stimuli on the time-dependent response of human Achilles tendon in vivo. It was shown for the first time, that heavy resistive ankle plantarflexion/ dorsiflexion exercises induced an immediate and significant decrease in Achilles tendon thickness (~15%). While thickness returned to pre-exercise levels within 24 hours, the recovery was exponential, with primary recovery occurring in less than 6 hours post-exercise. We proposed that such a diametral strain response with tensile loading reflects collagen realignment, Poison’s effects and radial extrusion of water from the tendon core. With unloading, the recovery of tendon dimensions likely reflects the re-diffusion of water via osmotic and/or inflammatory driven processes. Interestingly, prolonged walking was found to induce a similar diametral strain response. In subsequent studies, we demonstrated that eccentric exercise resulted in a greater reduction (-21%) in Achilles tendon thickness than isolated concentric exercise alone (-5%), despite a similar loading impulse. These novel findings, coupled with observations of a reduced diametral strain response with tendon pathology, highlight the importance of fluid movement to tendon function, nutrition and health. They also provide new insights into potential mechanisms underlying Achilles tendinopathy that impact rehabilitation strategies.

